# First Clinical Experience of a Novel Pulsed Solid-State Thulium:YAG Laser during Percutaneous Nephrolithotomy

**DOI:** 10.3390/jcm12072588

**Published:** 2023-03-29

**Authors:** Julius Bergmann, Clemens Mathias Rosenbaum, Christopher Netsch, Andreas J. Gross, Benedikt Becker

**Affiliations:** Department of Urology, Asklepios Hospital Barmbek, 22307 Hamburg, Germany

**Keywords:** percutaneous nephrolithotomy, thulium laser, urolithiasis, endourology, Mini-PCNL

## Abstract

Introduction: Laser lithotripsy during Mini-PCNL is one treatment option in urinary stone disease. In recent years, a new era in stone treatment has been initiated with the introduction of new pulsed thulium lasers. The aim of this study was to investigate the safety and efficacy of laser lithotripsy with a new pulsed solid-state thulium:YAG laser during mini-PCNL. Materials and methods: All patients, regardless of stone size, who were treated with a Mini-PCNL using the new pulsed thulium laser were prospectively enrolled. Operation times, stone size, laser time, and laser settings were noted. The stone-free rate was assessed postoperatively with sonography and either X-ray or computed tomography as a clinical standard. The complications were analyzed using the Clavien-Dindo classification. Results: A total of 50 patients with a mean age of 52 years were included. 31 (62 %) patients were male. The average stone size was 242.3 (±233.1) mm^2^ with an average density of 833 (±325) Hounsfield units. The mean operating time was 30.56 (±28.65) min, and the laser-on-time was 07:07 (± 07:08) min. The most commonly used settings were 0.4 J and 115 Hz (46 W). The mean total energy for stone ablation was 14,166 (±17,131) kJ. The total stone-free rate was 84 %, with an overall complication rate of 32% according to Clavien-Dindo (grade 1: n = 9, grade 2: n = 6, 3b: n = 1). In the group of patients with singular stones (n = 25), the stone-free rate was 88%. Summary: The new pulsed solid-state Thulium:YAG laser allows a safe and effective lithotripsy during Mini-PCNL. The stone-free rates were high regardless of stone size with a comparable low rate of complications.

## 1. Introduction

Percutaneous nephrolitholapaxy (PCNL) was first described by Fernström in 1976 [1]. Until today, the PCNL is considered as first-line therapy for renal calculi >2 cm [2]. For stone breakage during PCNL, different energy devices are available [3]. Rigid pneumatic and ultrasonic lithotripsy devices are frequently used. For pneumatic or ballistic lithotripsy, a rapid forward motion of a rigid projectile that produces a jackhammer effect on the target stone is used. However, different mechanisms exist to produce the ballistic effect depending on the company that offers this system. Pneumatic lithotripsy is more suitable for hard stones due to a very strong impulse. The ultrasonic lithotripsy works with less strong but more frequented impulses. This procedure is not suitable for hard stones, but the tissue defect is significantly smaller if the surgeon is careless [3]. A combined lithotrite device is also available and a further development includes the ability of suction during lithotripsy of fragmented stones [4].

As a second source of energy, lasers can be used for lithotripsy during PCNL. When using a laser either a fragmentation with high energy and low frequency or a dusting setting with high frequency and low energy can be applied to break the stone. However, other techniques such as popcorning, pop-dusting, or very fine dusting have been described as well [5]. Since 1995 the Holmium(Ho):YAG laser is the standard laser lithotripter for urinary calculi regardless of stone composition. The holmium laser uses white light that is emitted from a flashlamp to excite Holmium ions that are chemically bound to a YAG crystal. The interaction of the white light and the crystal results in the emission of photons with a characteristic wavelength of 2120 nm. This process repeats itself repetitively and is supported by a two-sided reflection by mirrors in the laser cavity which adds to holmium ions excitation, referred to as “laser pumping” [6]. Disintegration of stones was successful with this device, but the limitation of frequency and a distinct retropulsion were obvious drawbacks for this laser. Until a few years ago, thulium lasers could not be used for stone lithotripsy due to a continuous wave mode of the laser [7].

However, over the last years, new pulsed thulium (Tm) lasers have been developed and introduced into the armamentarium of laser lithotripsy (LL). In contrast to flashlamps, which are used in Ho:YAG lasers to excite the ions and therefore require special cooling to avoid excessive heat generation, the thulium ions in thulium fiber lasers (TFLs) or Tm:YAG lasers are excited directly via high-power laser diodes. The narrow-band excitation via laser diodes thus prevents excessive heat generation and energy efficiency is improved. In addition to the pulsed or cw mode, a wide range of different settings is also possible with the new thulium lasers (pulse energy, pulse frequency, and pulse width). 

The TFL uses various laser diodes as an energy source, which are operated with electrical current. The resulting laser emission, which emits at a wavelength of 1940 nm, is transmitted within a 10–30 m long active fiber doped with thulium ions and is further transmitted to the connected laser fiber. The TFL can operate at relatively high-power settings (up to 500 W) and theoretical frequencies of up to 2000 Hz, all while requiring only fan air cooling. 

The TFL has shown faster operation times with smaller stone fragments compared to Ho:YAG lasers both in preclinical studies and in clinical use. Another innovation in the field of thulium lasers was recently presented as a solid-state Tm:YAG laser. This laser can work in both pulsed mode and traditional continuous wave mode. This means that both stones and soft tissue can be treated effectively with this laser machine. With an application of up to 300 Hz, a pulse peak power of up to 1000 W, and a fully adjustable pulse width from 100–4750 μs, it is believed that it is possible to dust or fragment even harder stones in less time. Similar to the TFL, this pulsed Tm:YAG laser achieves laser pumping by electronically modulating diode lasers, however, uses a YAG crystal (instead of an active fiber) comparable to a Ho:YAG laser. The laser pumping excites thulium-electrons into higher-energy quantum states. The energy is focused by a fiber coupling lens and is then transmitted to the connected laser fiber. The resulting wavelength of 2013 nm is therefore located between the conventional holmium laser and the TFL [8]. The aim of the present study was to investigate the safety and efficacy of this new, yet not clinically tested, solid-state thulium:YAG laser during Mini-PCNL.T

## 2. Material and Methods

### 2.1. Patient Selection and Study Design

After obtaining institutional review board approval, a prospective study was performed to evaluate the safety, efficacy and stone-free rate (SFR) of Mini-PCNL utilizing a novel pulsed solid-state Thulium:YAG laser for lithotripsy. Fifty consecutive patients were prospectively enrolled between September 2020 and May 2021. Exclusion criteria were: age <18 years, concurrent other surgical treatment, and additional ureteral stones. 

Before surgical treatment, the following parameters were assessed: urine analysis and culture, a blood sample including serum creatinine and coagulation parameters, and radiology diagnostics with either kidney ureter bladder (KUB n = 13) X-ray or non-contrast computed tomography (NCCT n = 37). 

Stone characteristics such as size, location, and Hounsfield Units (HU) were documented. All laser settings (Joule (J), Hertz (Hz), Watt (W)), total energy, laser fiber size, and laser-on-time (LOT) were documented as well. 

### 2.2. Surgical Technique

All operations were performed by experienced endourologists. Patient positioning and percutaneous access were carried out in an analogous manner in all patients. After general anesthesia had been given, a 7 F multi-perforated double J catheter was inserted transurethral in the lithotomy position in order to retrogradely fill the renal pelvic system after insertion of a 22 F 3-way irrigation catheter.

All patients received intravenous antibiotic prophylactic with a second-generation cephalosporin (Cefuroxime), or according to the antibiogram.

After the patient had been placed in prone position, the lower calyx was punctured under combined ultrasound/fluorescence control, and the percutaneous tract was established. The tract dilation was carried out using a single-step dilation as described previously [2]. All renal calculi were disintegrated with a novel solid-state Thulium:YAG laser (RevoLix Hybrid Thulium Laser (HTL), LISA Laser products GmbH, Katlenburg, Germany). Three different fibers were used for lithotripsy (700 µm (n = 28), 550 µm (n = 17), and 300 µm (n = 5))(LISA Laser products GmbH, Katlenburg, Germany). The stone fragments were either removed passively using the “vacuum cleaner effect” (n = 45) or with an additional basket (n = 5). At the end of surgery, a 16-F nephrostomy catheter was inserted.

### 2.3. Outcome Measures

Operative time was measured as the time from puncture of the kidney until insertion of the nephrostomy. LOT, Hz, J, and W were automatically recorded by the laser device. The radiation dose (µGy/m^2^) and fluoroscopy time (s) were documented from the X-ray system and included the PCNL procedure as well as the previous ureteral stenting.

The complication rate was assessed with the Clavien-Dindo classification [9]. Patients were reassessed three months postoperatively with documentation of re-treatment rate and complications. The SFR was defined as no residual stones (<1 mm) at all and was proofed endoscopically during surgery and at postoperative day one with KUB (n = 30) or NCCT (n = 20). 

### 2.4. Statistical Analysis

Primary outcome variables included operative time and SFR. Secondary outcomes were LOT and complication rate. Patient data were expressed as mean ± standard deviation (SD) and a t-test was performed for statistical analyses. The significance level for all statistical analyses was *p* < 0.05. Statistical analyses were performed using SPSS version 20.0 (IBM Corp., Armonk, NY, USA).

## 3. Results

Of 50 patients who were included, 62% (n = 31) were male and 38% (n = 19) were female. Table 1 presents all patient and stone characteristics. The median age was 52 years with an average BMI of 28.4 (±7.4). The mean stone size was 242.3 (±233.1) mm^2^. Of those patients who received an NCCT (n = 37), the mean density was 830 HU (±195). Fifty percent (n = 25) of the patients had multiple stones. 

Postoperative outcomes are shown in Table 2. The mean LOT was 7:07 (±7:08) min with a mean total operative time of 30.56 (±17.5) min. In the subgroup of patients with single stones (n = 25), the mean total operative time was lower with 28.48 (±16.5) min. The mean total energy for stone ablation was 14,166 (±17,131) J. In 33 cases, dusting with high frequency (115 Hz) and low energy (0.4 J) was used for stone disintegration. 

The total SFR was 84% with an overall complication rate of 32% according to the Clavien-Dindo classification system (Grad 1 n = 9, Grad 2 n = 6, Grad 3b n = 1). The Clavien 3b complication (injury of the spleen) was however not directly linked to the PCNL. After the stone was removed, it was not possible to insert the nephrostomy through the PCNL tract due to a dislocation of the guidewire. While trying to place the nephrostomy tube with its inlay, it went upwards which could be seen during fluoroscopy. During this attempt to insert the nephrostomy, it stripped the spleen and a splenectomy was inevitable.

In the subgroup of patients with single stones, the stone-free rate was higher with 88% according to a similar complication rate. Overall, in eight patients a secondary treatment (Re-PCNL n = 2, Re-URS n = 6) due to residual stones was necessary.

## 4. Discussion

The key finding of our study was that the laser lithotripsy with the novel pulsed solid-state Thulium:YAG laser showed efficient fragmentation and dusting during Mini-PCNL. Until recently, the most frequently used laser for LL during Mini-PCNL was the Ho:YAG laser [10]. In the latest years, there was a shift from low-power to high-power holmium lasers with better modifications of the laser settings to dust urinary stones [11]. By modifying these parameters, a so-called “burst effect” for LL in urolithiasis was presented in 2016. It describes a set of laser pulses that follow each other in rapid succession. Despite the promising data, however, this effect could not prevail in a clinical setting [12]. In 2017, Elhilali et al. presented another innovation using certain Ho:YAG lasers with the “Moses Effect” [13]. This effect is currently being investigated in clinical studies and, in addition to LL, is also used for prostate enucleation. Large et al. postulated that enucleation using the Moses technology leads to improved vaporization and coagulation performance and the adenoma tissue can be separated more quickly from the prostate capsule [14]. Moreover, higher stone ablation rates with lower retropulsion could also be seen [13,15]. However, very fine dusting was not possible due to a limitation of the frequency [6,16].

Pulsed thulium lasers were currently established and showed better results in various in vitro experiments compared to Ho:YAG lasers [16,17,18]. Presently, there are two different types of pulsed thulium lasers available for clinical practice: TFLs and solid-state Tm:YAG lasers. However, only sparse data are available of these lasers in clinical settings [19]. In 2020, Enikeev et al. showed that the TFL leads to higher ablation speeds and therefore shorter operation times (OT) [20]. The same study group showed that the TFL provides effective and safe lithotripsy during ureteroscopy regardless of stone density for ureteral stones [21]. The high frequency with better dusting opportunities is one of the main advantages of the new pulsed thulium lasers generation. Ulvik et al. and Ryan et al. both underlined these results and noticed an outperformance of a pulsed TFL with regards to SFR and OT compared to a Ho:YAG laser [22,23]. In contrast to these publications, there are also studies that describe an equal effectiveness between holmium and new thulium lasers, especially with regard to the pulse-modulated holmium laser [24,25]. In our study, we achieved an overall SFR of 84% for all stone sizes which is line with the current literature that shows a SFR between 72.6–100% for the Mini-PCNL [26]. However, in the mentioned papers, no data of experience was documented which could explain the wide range of SFRs. Only few studies assessed the clinical efficacy of TFL during Mini-PCNL with comparable SFR to ours, but no study investigated the solid-state Thulium:YAG laser yet [4,19,27,28]. In this study, the dusting mode (115 Hz, 0.4 J) as well as the fragmentation mode (10 Hz, 1 J) showed a high efficacy in stone breakage. Irrespective of the used regime, the pulse width was set to 100%.

The LOT for fragmenting was shorter compared to dusting or a combination of both techniques. However, this difference was not evident in the overall surgery time because the higher energy per pulse with low frequency meant more stone passages due to bigger fragments and less dust, which equalized the shorter LOT in the overall surgery time. The way a stone is processed during Mini-PCNL is therefore not relevant to the total operation time, which is an important finding with regards to the new generation of thulium lasers. The higher peak power of this laser might be superior to a TFL for the treatment of harder stones; however, this has to be investigated in further studies.

A subgroup analysis of singular stones of our cohort showed faster OT and less complications compared to multiple stones. However, regardless of the heterogeneity in our cohort, we showed a high SFR with the HTL during Mini-PCNL.

In the aforementioned study of Enikeev et al., the OT was 23.4 min for the Mini-PCNL while using the TFL [19]. Our OT was slightly higher, but still lower compared to the literature with a mean of 30.56 min. This might be since both lasers are of comparable quality. Furthermore, both studies took place in departments with a high number of yearly performed Mini-PCNLs that goes in line with a high experience of the surgeons. 

The fact that we did not perform an NCCT in every patient might lead to radiological measurement errors of the stone size and the measured SFR [29]. The SFR was defined as no residual stones (<1 mm) at all and was proofed endoscopically during surgery and at postoperative day one with KUB (n = 30) or NCCT (n = 20). Hence, it is well known that an NCCT has a higher sensitivity and specificity in the diagnosis of kidney and ureter stones than a KUB [30,31,32]. Still, according to our clinical standards, we only perform a postoperative NCCT for stones >3 cm, difficult anatomy, multiple stones, known uric acid stones, or difficult surgical conditions with limited visibility. This is the reason for the inconsistency of the postoperative measurement of the SFR. Gokce et al. showed the lower accuracy of plain radiograph compared to the NCCT for residual fragments after PCNL [33], but this was not within the scope of this study to detect diagnostic differences between the two different imaging modalities. Our experience to perform an NCCT at postoperative day 1 often supposedly shows a remaining stone mass. This leads to a second PCNL or ureteroscopy, but in the majority of these cases, the intraoperative view shows stone dust and no rigid stone mass to basket. A better way for postoperative SFR assessment would be to perform an NCCT after approximately four weeks. Conversely, an NCCT performed this late is difficult to explain ethically. Furthermore, in our opinion a decision for a re-do surgery should be made within the first postoperative days as the patients should not leave the hospital with a nephrostomy tube inserted. 

Another limitation is the single arm study design. However, the intention of the study was to show the safety and efficacy of this novel laser that has not been published before. For this aspect, no control group is necessary to test the mentioned parameters [17,19]. Furthermore, this study did not investigate on the stone composition. It is well known that the ablation speed depends on the hardness of the stone, but the HU in the NCCT, which we used in this study, is also suitable for this. This study has shown the first cohort of patients treated with the novel pulsed solid-state HTL during Mini-PCNL.

## 5. Conclusions

To the best of our knowledge, this is the first study that investigated the pulsed Tm:YAG laser for laser lithotripsy during Mini-PCNL. It was shown that it is a highly efficacious laser for the treatment of renal calculi during Mini-PCNL with lower operation times compared to the literature and with a comparable complication rate.

## Figures and Tables

**Table 1:** **a**—Patient characteristics (n = 50). **b**—Stone characteristics.

(**a**)		
Patients (n)	50 (m = 31, f = 19)	
Age, (years)	52 ±13.67 (21–81)	
Body mass index, (BMI)	28.4 ± 7.4 (15.79–52.86)	
Number of stones (n)	1.86 ± 1.24 (1–8)	
Stone size, (mm^2^)	242 ± 233 (30–1262)	
Stone density, (Hounsfield Units)	830 ± 325 (418–1572)	
Data presented as mean ± SD (range)		
(**b**)		
Stone Location	n	(%)
Renal pelvis	17	34.0
Lower pole	8	16.0
Middle pole	3	6.0
Upper pole	2	4.0
Multiple location	20	40.0
Multiple location but not lower pole	4	8.0
Total	50	100.0

Data presented as n (%).

**Table 2:** Intra- and postoperative parameters.

Intraoperative Parameters	
Surgery time (min)	30.56 ±17.5 (15–64)
Stone-free rate	84%
Laser, Total Energy [J]	14,166 ± 17,131 (38–71,989)
Frequency (Hz)	81 ± 44 (10–200)
Joule	0.4 ± 0.2 (0.01–1)
Laser on time (min)	7:07 ± 07:08 (2–38:44)
Hospital stay	2.8 ± 1.56 (1–11)
Fluoroscopy time (DJ insertion + PCNL) (s)	48.78 ± 35.97 (3–164)
Radiation dose (DJ insertion + PCNL) (µg/m2)	80.93 ± 72.61 (4.57–376.59)
Postoperative Complications	Total
Clavien grade I	
clot retention	2
fever	5
transient creatinine elevation (>0.5 mg/dL)	2
Clavien grade II	
Urinary tract infection	4
Blood transfusion	1
Urine leakage < 24 h	1
Clavien grade 3 b	
Neighboring organ injury–splenectomy	1
total	n = 16 (32%)

Data presented as n (%); Data presented as mean ±SD (range).

## Data Availability

Data is unavailable due to privacy.
